# Molecule-displacive ferroelectricity in organic supramolecular solids

**DOI:** 10.1038/srep02249

**Published:** 2013-07-22

**Authors:** Heng-Yun Ye, Yi Zhang, Shin-ichiro Noro, Kazuya Kubo, Masashi Yoshitake, Zun-Qi Liu, Hong-Ling Cai, Da-Wei Fu, Hirofumi Yoshikawa, Kunio Awaga, Ren-Gen Xiong, Takayoshi Nakamura

**Affiliations:** 1 Research Institute for Electronic Science, Hokkaido University, Sapporo 001-0020, Japan; 2 Ordered Matter Science Research Center, Southeast University, Nanjing 211189, China; 3 Research Center for Materials Science and Department of Chemistry, Nagoya University, Furo-cho, Chikusa-ku, Nagoya 464-8602, Japan; 4 These authors contributed equally to this work.

## Abstract

Ferroelectricity is essential to many forms of current technology, ranging from sensors and actuators to optical or memory devices. In this circumstance, organic ferroelectrics are of particular importance because of their potential application in tomorrow's organic devices, and several pure organic ferroelectrics have been recently developed. However, some problems, such as current leakage and/or low working frequencies, make their application prospects especially for ferroelectric memory (FeRAM) not clear. Here, we describe the molecule-displacive ferroelectricity of supramolecular adducts of tartaric acid and 1,4-diazabicyclo[2.2.2]octane *N*,*N*′-dioxide. The adducts show large spontaneous polarization, high rectangularity of the ferroelectric hysteresis loops even at high operation frequency (10 kHz), and high performance in polarization switching up to 1 × 10^6^ times without showing fatigue. It opens great perspectives in terms of applications, especially in organic FeRAM.

Ferroelectrics are an interesting class of materials, which have been explored and found a variety of technical applications^1^,^2^. In recent years, a great number of research activities have been focused on designing molecule-based ferroelectric materials^3^,^4^,^5^,^6^,^7^. Ferroelectricity in pure organic solids^8^ has been achieved only in a few π···π-stacked or hydrogen-bonded supramolecular complexes^9^,^10^. They consist of one-dimensional chains of electron/proton donors and acceptors, along which electron/proton transfer may induce spontaneous electric polarization (*P*_s_) via a neutral-to-ionic (NI) process (D^0^A^0^D^0^A^0^ → D^δ+^A^δ–^D^δ+^A^δ–^)^10^,^11^,^12^. These new ferroelectrics exhibit attractive properties, such as large *P*_s_ and dielectric constants^9^,^10^. However, some problems, such as current leakage in the charge-transfer complexes^10^,^11^,^13^ and/or low working frequencies^12^ limit their practical utilization. Searching for new organic ferroelectrics and their characterization remain a challenge. Here, we describe excellent ferroelectric properties of new supramolecular adducts of 1,4-diazabicyclo[2.2.2]octane *N*,*N*′-dioxide (dabcodo) and L(+) or D(–)-tartaric acid (LTa or DTa) (Fig. 1a), in which the spontaneous polarization originates from reorientation of O–H bonds induced by the relative displacement between the two molecular sublattices (Fig. 2).

## Results

### Structural phase transition

**Figure 1:**
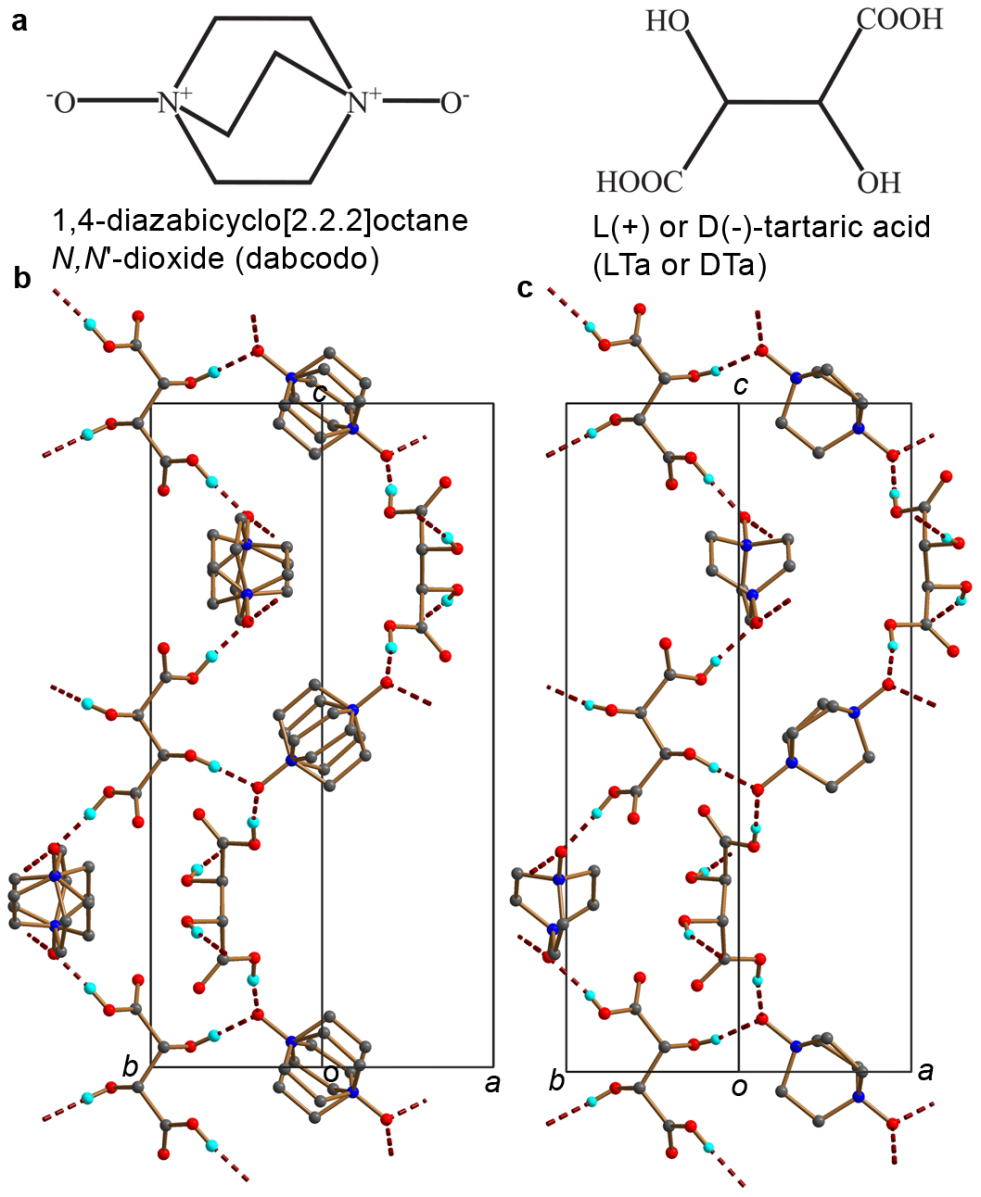
Chemical formulae and crystal structures of the adduct of L(+) or D(–)-tartaric acid (LTa or DTa) and 1,4-diazabicyclo[2.2.2]octane *N*,*N*′-dioxide (dabcodo). (a) Chemical formulae of the organic molecules used in construction of the adducts. (b) The structure of the high-temperature phase of dabcodo-LTa. (c) The structure of the low-temperature phase of dabcodo-LTa. The dotted lines indicate the intermolecular O–H···O Hydrogen bonds.

Dabcodo-LTa was found to undergo a phase transition at around 254 K by using thermal analysis (Fig. 3). In the high-temperature phase (HTP), it crystallizes in the tetragonal space group *P*4_1_2_1_2 (CCDC 921381) (see Supplementary Fig. S4 and Table S1). Accordingly, dabcodo-DTa has an enantiomeric space group *P*4_3_2_1_2 (CCDC 921384). A characteristic feature of this structure is the head-to-tail hydrogen-bonded chains, with an O···O distance of 2.547(2) Å. These chains are further linked side-to-side, forming a three-dimensional hydrogen-bonded network (Fig. 1). The centres of the molecules are located on the two-fold axes in the [1 1 0] and [1 –1 0] directions, and two O–H^acid^···O or O–H^alc^···O Hydrogen bonds are symmetry-equivalent.

**Figure 2:**
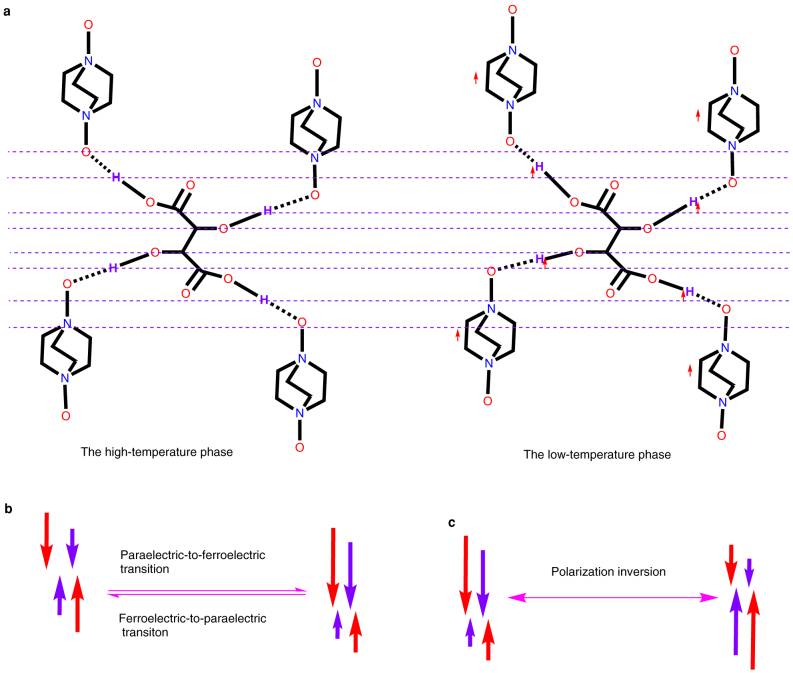
Schematic drawing of ferroelectricity achieved in organic supramolecular adducts. The spontaneous polarization originates from reorientation of the O–H bonds, which is induced by the relative displacement between the dabcodo sublattice and the tartaric acid sublattice. (a) Schematic drawing of how the relative displacement between the sublattices is connected to the reorientation of the O–H bonds. The arrows indicate the displacement in the direction of *c*-axis. (b) Schematic drawing of how the reorientation of the O–H bonds in one molecule induces spontaneous polarization. The red arrows denote components of dipole moments of the O–H^acid^ bonds in the direction of the polar axis; the purple arrows denote components of dipole moments of the O–H^alc^ bonds in the direction of the polar axis. (c) Schematic drawing of how the polarization inversion is achieved.

The low-temperature phase (LTP) becomes the polar space group *P*4_1_ (CCDC 921382) (see Supplementary Fig. S5). The structural refinement provided evidence for the formation of 180° ferroelectric domains. The initial refinement converged to *R*_1_/*wR*_2_ = 0.17/0.48. With the twin law of 180° rotation symmetry in the direction [1 0 0] suggested by *ROTAX*^14^, the final refinement converged to *R*_1_/*wR*_2_ = 0.024/0.060. The hydrogen bond geometries approximate those in the HTP. However, the *c*-direction components of the distances between the donor and acceptor atoms for the two O–H^acid^···O or O–H^alc^···O Hydrogen bonds show significant difference, 0.1024 Å and 0.3281 Å respectively (see Supplementary Table S2). Those differences correspond to a relative displacement of 0.1344 Å along the *c*-axis between the two molecular sublattices (Fig. 2a). In the structures at both temperatures, the two acidic protons located from the differential Fourier map remain attached to the tartaric acid molecule, the structural parameters of the carboxyl groups being comparable to those in the neutral molecule^15^. The formation of strong Hydrogen bonds without discernible proton transfer is revealed by the vibrational spectra. In the room-temperature infrared (IR) absorption spectra (KBr pellet) (see Supplementary Fig. S8), the OH^acid^-stretching bands^16^ become wider and stronger, and show large red shifts (≈ 170 cm^−1^) upon formation of the adduct, whereas the stretching band of the C = O bonds just shows a small red shift.

**Figure 3:**
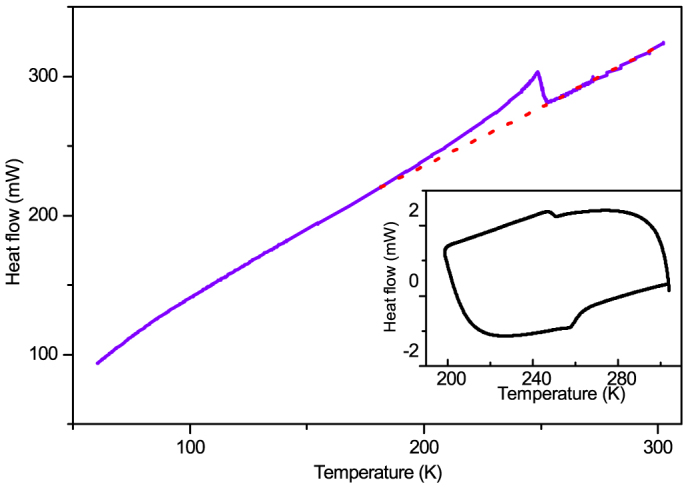
Heat capacity of dabcodo-LTa as a function of temperature. A subtle anomaly below 150 K is discernible. Inset: the DSC plot for dabcodo-LTa, showing the reversibility of the transition. The crystals used in the measurements have sizes of ≈ 3 mm.

The halving of the number of symmetry elements upon the transition (Aizu notation^17^ 422F4) indicates a second-order phase transition^18^ (for analysis of spatial symmetry change, see Supplementary Fig. S6). Thermal analysis reveals the continuity as a step-like anomaly in the *C_p_* and DSC curves (Fig. 3). The transition enthalpy of 790 J/mol corresponds to an entropy change of 2.8 J mol^–1^ K^–1^, which is considerably smaller than *R*ln2 (*R* is the gas constant). It appears the ordering of the dabcodo takes place over a much wider temperature range, de-emphasizing the order–disorder character of the transition. The continuity also shows in the temperature dependence of the lattice parameters (see Supplementary Fig. S7). The *a*-axis shows linear thermal expansion; the expansion of the *c*-axis is continuous, but with an inflection point at around 254 K, which is a sign that the transition results from a structural change in the direction of the *c*-axis.

### Ferroelectric properties

**Figure 4:**
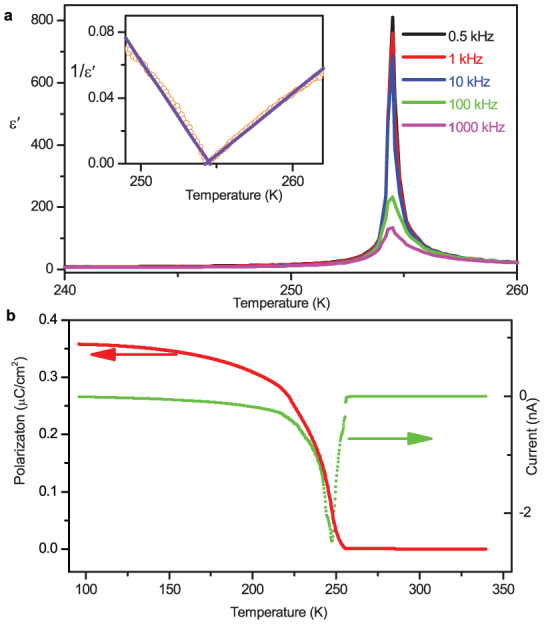
Dielectric and polar responses for the transition at 254 K. (a) The temperature dependence of the real parts of the complex dielectric constants in the *c* direction. Inset: Plot of 1/*ε*′ vs temperature. (b) The temperature dependence of the spontaneous electric polarization *P*_s_ calculated by integrating the pyroelectric current measured upon heating.

The ferroelectric nature of the transition is evident from the large dielectric constant anomalies in the *c*-direction at the Curie temperature *T*_c_ = 254.5 K (Fig. 4a). In the vicinity of the critical temperature, the dielectric response shows Curie–Weiss behaviour, *ε*′ = *C*_p_/(*T* – *T*_0_) (*T* > *T*_c_) or *C*_f_/(*T*_0_′ – *T*) (*T* < *T*_c_), with Curie constants *C*_p_ = 138 K and *C*_f_ = 72 K, and Weiss temperatures *T*_0_ ≈ *T*_0_′ = 254.5 ± 0.2 K. The *C*_p_/*C*_f_ ratio of 1.92 is quite close to the theoretical value (*C*_p_/*C*_f_ = 2) expected for a second-order phase transition.

The larger *ε*′ in the vicinity of *T*_c_ is suggestive of a larger *P*_s_, as a proper ferroelectric. We determined *P*_s_ by integrating the pyroelectric current (Fig. 4b). As expected, *P*_s_ is nonzero below *T*_c_; it vanishes above *T*_c_. The observed continuity of *P*_s_ is in accordance with the character of a second-order transition.

**Figure 5:**
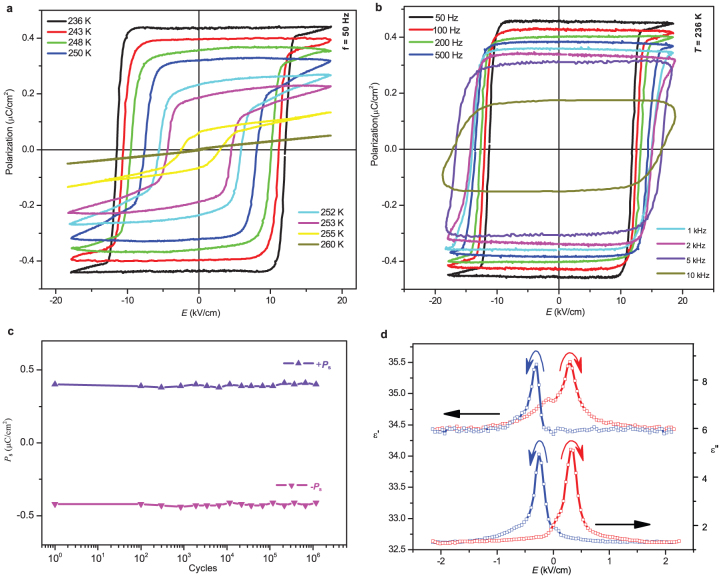
Properties of polarization switching for dabcodo-LTa. (a) Temperature dependence of *P*–*E* hysteresis loops at 50 Hz. (b) Frequency dependency of *P*–*E* hysteresis loops at 236 K. (c) Result of the ferroelectric fatigue measured at 243 K at a frequency of 50 Hz. No fatigue was observed. (d) Bias field dependence of the complex dielectric constant of dabcodo-LTa at 240 K.

The polar response was confirmed by the polarization–electric field (*P*–*E*) hysteresis behaviour. The linear *P*–*E* dependence at 260 K in Fig. 5a is consistent with the paraelectric behaviour. Perfect loops are observed in the steady ferroelectric phase below 250 K. The *P*_s_ has the same order of magnitude as those of the phenazine-based cocrystals (*P*_s_ = 0.7–0.8 μC/cm^2^)^8^ as well as Rochelle salt series (0.25–1 μC/cm^2^)^19^, but smaller than that of croconic acid^20^. This is because only one component of the polarity of the O–H bonds contributes to *P*_s_ in dabcodo LTa, as will be discussed below; while in corconic acid, the proton transfer contributes directly to *P*_s_. *P*_r_ reduces with an increase in the applied frequency (Fig. 5b). It is interesting to find that, at a frequency even as high as 10 kHz, the loops retain high rectangularity. This is the highest operating frequency reported so far in organic ferroelectrics^12^,^20^,^21^. A preliminary study on the ferroelectric fatigue properties shows that *P*_s_ remains unchanged after 1 × 10^6^ switching operations (Fig. 5c).

Analogous to the *P*–*E* hysteresis behaviour, the DC electric field dependence of the complex dielectric constant shows ‘butterfly’-shape hysteresis loops, which are more pronounced for the imaginary part (Fig. 5d). This is further evidence for the appearance of switchable ferroelectric domains.

### Deuteration effects

**Figure 6:**
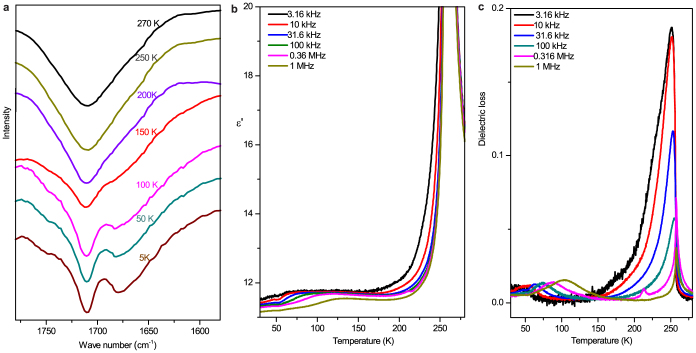
IR spectra and dielectric evidence for the order-disorder transition of the O–H^acid^ bonds below 150 K in Dabcodo-LTa. (a) Thermal variation of the C = O stretching band. (b) The temperature dependence of the real parts of the complex dielectric constants, showing wide temperature dispersion below 150 K. The peak values are far smaller than those at the ferroelectric transition temperature of 254.5 K. (c) The temperature dependence of the dielectric losses, showing wide temperature dispersion below 150 K.

To determine the role played by the hydrogen bonds, we prepared deuterated dabcodo-LTa (CCDC 921386, 921387), and only a negligible negative deuteration effect on *T*_c_ was observed in the DSC and dielectric measurements (see Supplementary Figs S3 and S9). It appears that no collective proton transfer is involved in the transition. However, variable-temperature dielectric and IR spectra reveal that an incipient acidic proton transfer has already taken place above 150 K in dabcodo-LTa. Below 150 K, the C = O stretching vibration band at 1710 cm^–1^ splits into two peaks without shift of the frequency (Fig. 6a). This can be ascribed to one of three possibilities: incipient collective acidic proton transfer along the hydrogen-bonded chains, splitting of the overlapped peaks because of crystallographically distinguishable sites for the two identical C = O vibrations, or the ordering of two types of chemically nonequivalent disordered O–H^acid^ bonds. The dielectric spectra support the latter case by dielectric anomalies dispersing in a wide temperature range from 150 K to 60 K (Figs. 6b and 6c), which points to an order–disorder transition of dipoles. The dispersion was confirmed not to be associated with a phase transition by the smooth *C_p_* curve below 150 K (Fig. 3), and by the crystal structure determination at 83 K (CCDC 921383). Since no dielectric anomaly corresponding to the incipient proton transfer was observed (Figs. 6b and 6c), the process may take place in the HTP. It should be noted that, replacing the H atoms with D atoms may affect the dielectric response below 150 K. However, the effect is indiscernible from the dielectric spectra measured on powder-pressed samples (see Supplementary Fig. S9), because the anomalies are too small to be visible.

## Discussion

Hydrogen-boned ferroelectrics are characterized by large deuteration effects on *T*_c_. The recently developed organic ferroelectrics, such as croconic acid^20^ and phenazine-based cocrystals^8^, can be regarded as this type. They are realized by proton transfers along hydrogen bonds, including collective proton transfer^22^, incipient proton transfer^23^ and proton tautomerism^20^, and correspondingly exhibit large deuteration effects^24^. Considering no significant deuteration effect and the obvious relative molecular displacement upon the transition in dabcodo-LTa, we postulate the ferroelectric mechanism as a molecule-displacive type, different from the hydrogen-boned type (Fig. 2). Because of the strong hydrogen-bond interactions, the relative displacement of the two sublattices induces the reorientation of both the O–H^acid^ bonds and the O–H^alc^ bonds between two asymmetric potential minima, thus generating *P*_s_. Below 150 K, both the displacive and the incipient proton transfer may contribute to *P*_s_ in dabcodo-LTa.

The intrinsically fast and low-fatigue polarization switching is consistent with the displacive-type mechanism, and such ferroelectric switching characteristics is highly desired for FeRAM. For nonvolatile RAM applications, ferroelectric polymers and oligomers [e.g. poly(vinylidene fluoride) (PVDF), poly(vinylidene fluoride-trifluoethylene) (P(VDF-TrFE)), and so on], have been studied intensively due to their low cost, flexibility and the possible multilayer structure^21^,^25^,^26^. Although their switching time is considerably short, being of 10^–6^–10^–8^ second order at room temperature if the applied field is sufficiently high, their hysteresis measurements can be made practically at low frequencies (1–10 Hz) because of the critical heat generation associated with hysteresis loss^21^. In that sense, despite relatively low Curie temperature, dabocdo–LTa opens great perspectives in terms of applications, especially in organic FeRAM.

The role of H atoms in hydrogen-bonded ferroelectrics has been the subject of debate because of the difficulty of diffraction techniques reliably determining their sites in crystals. For example, phase transitions in Rochelle salt^19^ exhibit the character of a mixture of displacive and order-disorder^27^,^28^, while the order–disorder features have not been clarified crystallographically yet^29^,^30^. Applying the finding in dabcodo-LTa to the transitions, the mechanism could be better understood in the framework of the theory of Mason^19^. The formation of dipoles can be understood as an incipient proton transfer along the Hydrogen bonds. Qualitatively, the order–disorder change of the dipoles may take place cooperatively with displacement upon the transitions. The Mason model ignored the contribution of the displacement to *P*_s_, and as such the expected proton transfer was not observed. The lack of discernible proton transfer may be due to another fact, namely that the incipient proton transfer affects the structure insignificantly, while in dabcodo-LTa it affects the C = O stretching vibration to an observable extent.

In summary, we have successfully developed a new type of organic ferroelectric based on supramolecular adducts. Because of their high performance in polarization switching and the availability of large high-quality single crystals, the adducts will be attractive candidates for use of organic materials as ferroelectric memories. The clear ferroelectric mechanism may contribute to an understanding of the physics in hydrogen-bonded ferroelectrics.

## Methods

### Crystal growth

The supramolecular adducts were obtained as crystals up to 2 cm in size (see Supplementary Fig. S1) by evaporation of an aqueous solution of stoichiometric amounts of dabcodo^31^ and L(+) or D(–)-tartaric acid at room temperature (see Supplementary information). The bulk phase purity was verified by powder XRD (see Supplementary Fig. S2).

### Synthesis of deuterated dabcodo-LTa

L(+)-tartaric acid (1.50 g, 10 mmol) was neutralized with KOH (1.12 g, 20 mmol) in D_2_O (50 g). The solution was refluxed for 16 h under a N_2_ atmosphere. After cooling to room temperature, a crystal was taken out for IR examination. No OH^alc^-stretching vibration peak was observed. Then, a HBF_4_ solution (42%) (4.18 g, 20 mmol) was added to the mixture, and the mixture was sufficiently stirred. After filtration, solid dabcodo (1.44 g, 10 mmol) was added. The resulting solution was allowed to evaporate at room temperature. After 16 hours, the precipitated crystals were collected and dried in vacuum. Although the theoretical degree of deuteration reaches about 95% with this procedure, and correspondingly, the IR spectra show two strong OD^alc^-stretching vibration peaks at 2469 and 2438 cm^–1^, the OH^alc^-stretching vibration peaks (come from undeuterated species) remain strong (see Supplementary Fig. S8).

### Physical properties measurement

For electronic measurements, single-crystal plates (0.37–0.5 mm thick and 10–50 mm^2^ in area) deposited with silver-conducting glue were used. The complex dielectric constants were measured in the frequency range 0.5–1000 kHz, using a Tonghui TH2828A or an Agilent 4294 Impedance Analyzer with an applied electric field of 0.5 V. The dielectric measurements at a frequency of 1 kHz with a DC bias field ranging from –400 V to +400 V were performed using a Novocontrol Alpha-A High Performance Frequency Analyzer. The *P*–*E* hysteresis loop was recorded on a Sawyer–Tower circuit, Precision Premier II (Radiant Technologies, Inc.). The ferroelectric fatigue test was performed with the same conditions as in the measurement of the *P*–*E* hysteresis loop. The temperature dependence of spontaneous polarization was calculated by integrating the pyroelectric current. The pyroelectric current was measured using an electrometer (Keithley 6517B) under zero electric field in a warming process at a rate of 10 K/min after carrying out a poling procedure with an electric field gradient of 500 V/cm. Infrared spectra (KBr pellet) were recorded on a NICOLET 6700 FT-IR spectrometer, equipped with an Oxford He Cryostat temperature-controlling system. Specific heat analyses were carried out using a Quantum Design PPMS. DSC measurements were performed using a Rigaku Thermal series instrument.

## Additional Information

**Accession Codes**: The structures have been deposited at the Cambridge Crystallographic Data Centre (deposition numbers: CCDC 921381 – 921387)

## Author Contributions

H.Y.Y. prepared the samples. Y.Z., Z.Q.L., H.L.C. and D.W.F. characterized the electric properties. K.K. and M.Y. measured the IR spectra. H.Y. and K.A. measured the heat capacity. R.G.X. wrote most of the manuscript. S.I.N. and T.N. designed and directed the studies and contributed to the writing of the manuscript.

## Supplementary Material

Supplementary Information Supplementary Infomation
