# Supplementary material for: Molecule-displacive ferroelectricity in organic supramolecular solids
Source: Sci Rep. 2013 Jul 22;3:2249. doi: 10.1038/srep02249 (PMC3718360; doi:10.1038/srep02249)
Supplement: Supplementary Information — Supplementary Infomation [file srep02249-s1.pdf]

Molecule-displacive ferroelectricity in organic supramolecurlar solids

Heng-Yun Ye[1,2], Yi Zhang[2], Shin-ichiro Noro[1]*, Kazuya Kubo[1], Masashi Yoshitake[1], Zun-Qi Liu[1], Hong-Ling Cai[2], Da-Wei Fu[2], Hirofumi Yoshikawa[3], Kunio Awaga[3], Ren-Gen Xiong[2]*, Takayoshi Nakamura[1]*

[1]Research Institute for Electronic Science, Hokkaido University, Sapporo 001-0020, Japan.

[2]Ordered Matter Science Research Center, Southeast University, Nanjing 211189, China.

[3]Research Center for Materials Science and Department of Chemistry, Nagoya University, Furo-cho, Chikusa-ku, Nagoya 464-8602, Japan.

A. Characterization

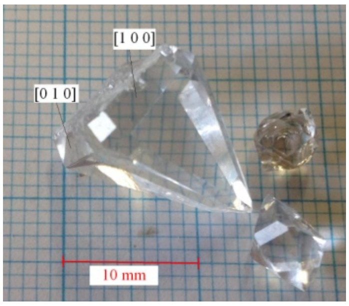

Figure S1 | A picture of crystals of dabcodo-LTa.

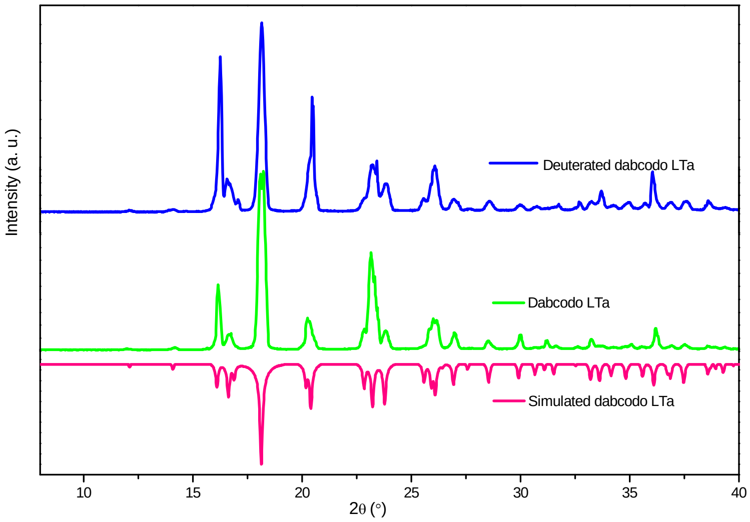

Figure S2 | Patterns of PXRD of the bulk phase of dabcodo-LTa and its deuterated specimen. There are several peaks missed in the sample PXRD. This is probably due to too weak intensity.

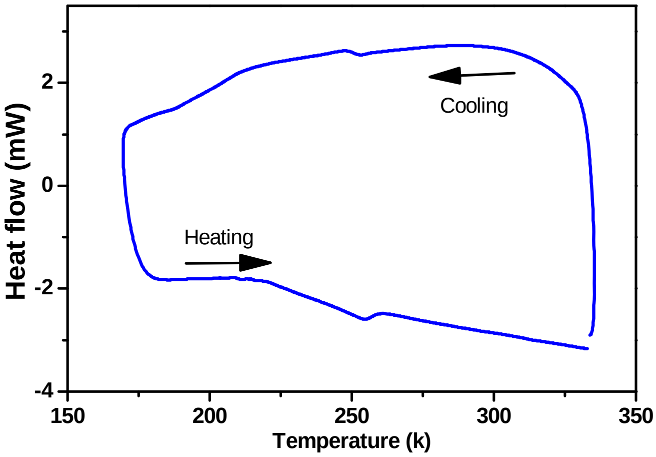

Figure S3 | The DSC result of deuterated dabcodo-LTa. The samples used in the measurements are crystals with sizes of ≈ 1.5 mm. For deuterated and non-deuterated dabcodo-LTa, the thermal anomalies depend on the sizes of the used crystals. The larger the crystals, the more obvious the anomalies. For powder samples, almost no anomalies can be observed.

B. Crystallographic Information

Table S1. Crystal data of dabcodo-LTa and its deuterated specimen in the HTP and LTP.

	dabcodo-LTa	dabcodo-LTa	Deuterated dabcodo-LTa	Deuterated dabcodo-LTa
Chemical formula	$C_{10}H_{18}N_2O_8$	$C_{10}H_{18}N_2O_8$	$C_{10}H_{14}D_4N_2O_8$	$C_{10}H_{14}D_4N_2O_8$
M_r	294.26	294.26	298.29	298.29
Crystal system, space group	Tetragonal, $P4_12_12$	Tetragonal, $P4_1$	Tetragonal, $P4_12_12$	Tetragonal, $P4_1$
Temperature (K)	293	138	293	133
a, c (Å)	7.7810 (11), 21.320 (4)	7.7364(11), 21.205 (4)	7.7858 (11), 21.316 (4)	7.7424 (11), 21.222 (4)
V (Å^3)	1290.8 (4)	1269.2 (4)	1292.1 (4)	1272.1 (4)
Z	4	4	4	4

Table S2. The components in the *c* direction of distances between the donor and acceptor atoms in dabcodo-LTa. The experimental errors were not included in the calculation.

Hydrogen bond	d^c_{D-A} (Å) (in the HTP)	d^c_{D-A} (Å) (in the LTP)
O–H^{acid}···O	1.8197	1.8966
		1.7942
O–H^{alc}···O	1.0266	1.1379
		0.8098

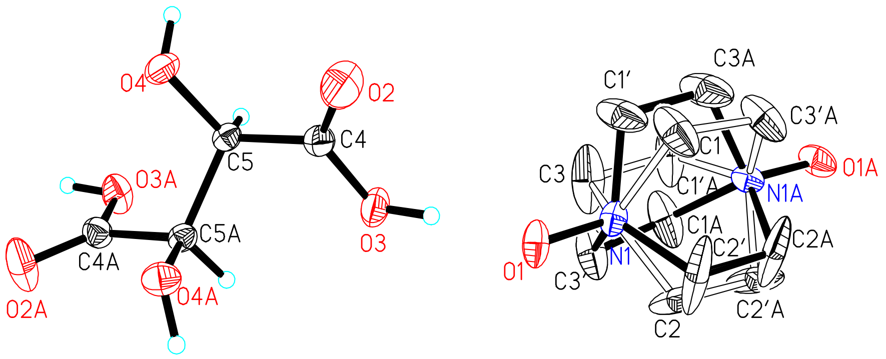

Figure S4 │ Molecular configuration of dabcodo-LTa with the atomic numbering scheme at 20 °C. Displacement ellipsoids are drawn at the 30% probability level. Atoms with suffix A were generated by a symmetry operation of the two-fold axis passing through the centres of the molecules. H atoms on dabcodo are omitted for clarity.

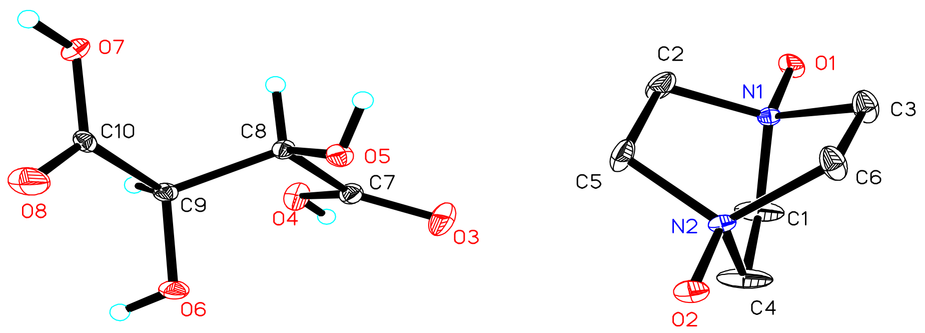

Figure S5 │ Molecular configuration of dabcodo-LTa with the atomic numbering scheme at –135 °C. Displacement ellipsoids are drawn at the 30% probability level. H atoms on dabcodo are omitted for clarity.

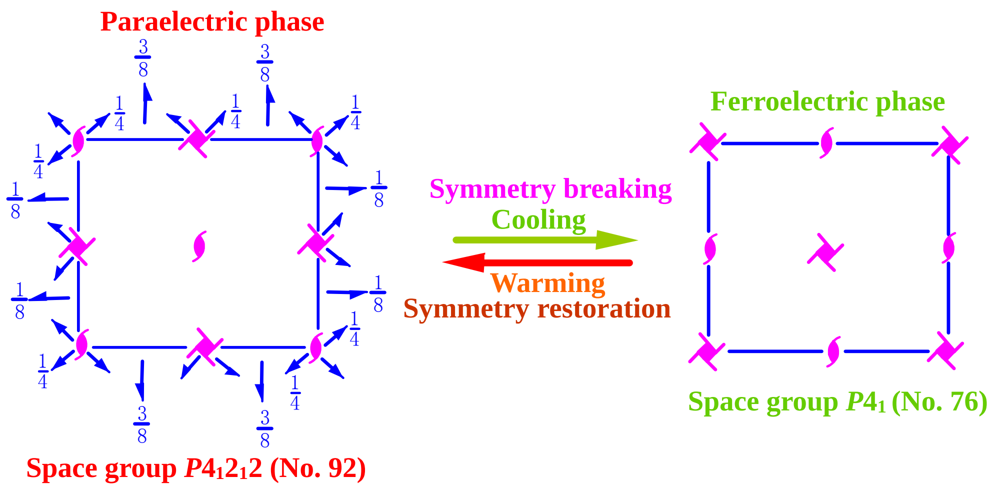

Figure S6 │ Spatial symmetry change between the paraelectric phase ($P4_12_12$) and the ferroelectric phase ($P4_1$) in dabcodo-LTa.

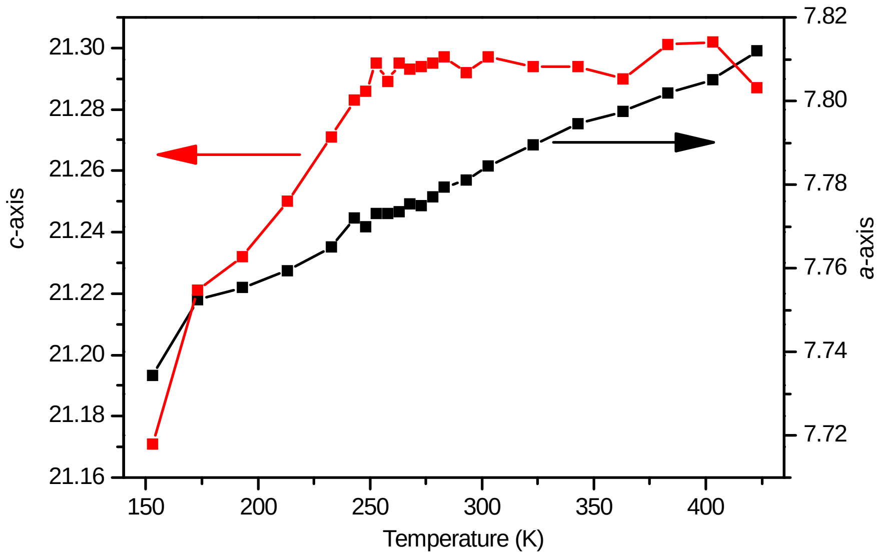

Figure S7 │ Evolution of the lattice parameters as a function of temperature.

C. IR Spectra

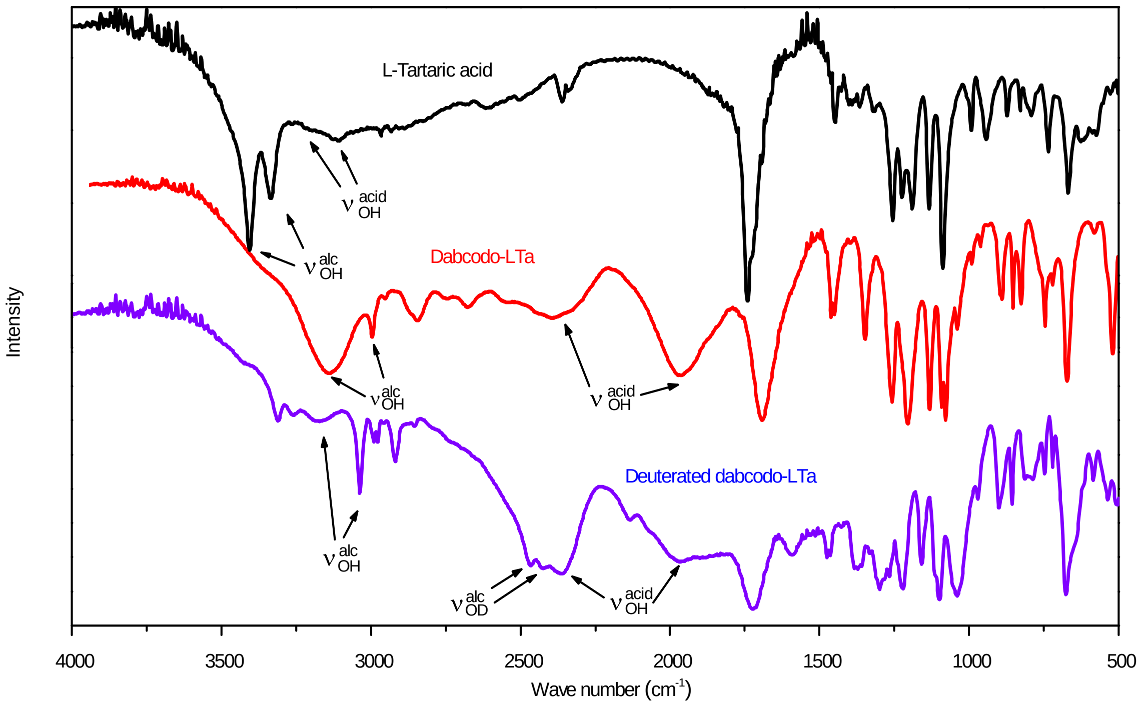

Figure S8 │ IR spectra of dabcodo-LTa, deuterated dabcodo-LTa, the starting material L-tartaric acid at room temperature (for the assignment of the $\nu_{\mathrm{OH}}^{\mathrm{alc}}$ and $\nu_{\mathrm{OH}}^{\mathrm{acid}}$ in tartaric acid, see Ref. 16).

D. Dielectric spectra

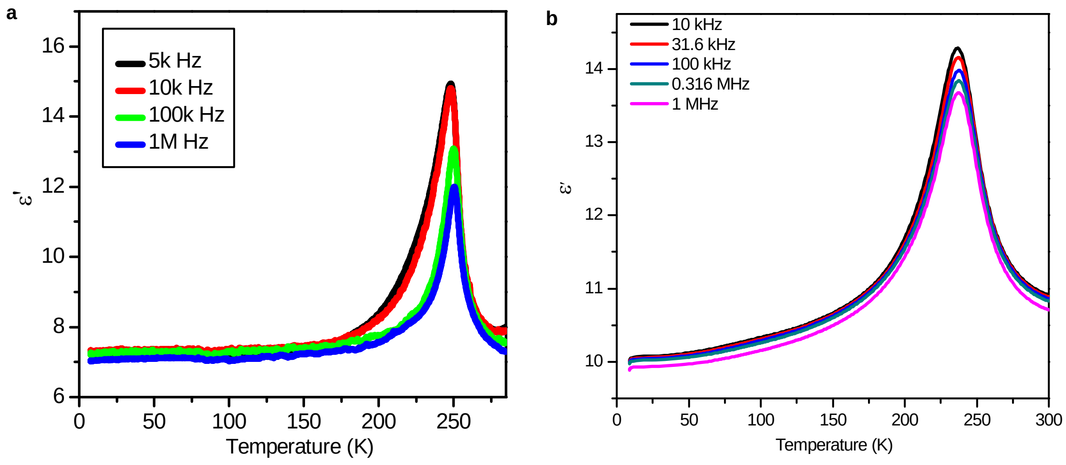

Figure S9 │ Comparison of the dielectric response of deuterated dabcodo-LTa and nondeuterated dabcodo-LTa, showing a minor negative deuteration effect on T_c at around 254 K. **a**, The temperature dependence of the real parts of the complex dielectric constants of nondeuterated dabcodo-LTa measured on a powder-pressed pellet. **b**, The temperature dependence of the real parts of the complex dielectric constants of deuterated dabcodo-LTa measured on a powder-pressed pellet.
